# Laparoscopic repair of the vaginal cuff dehiscence: Dehiscence occurring after the first sexual intercourse after the laparoscopic modified radical hysterectomy

**DOI:** 10.1002/ccr3.1906

**Published:** 2018-11-05

**Authors:** Yukio Suzuki, Yuichi Imai, Naho Ruiz‐Yokota, Etsuko Miyagi

**Affiliations:** ^1^ The Department of Obstetrics and Gynecology Yokohama City University Graduate School of Medicine Yokohama Japan

**Keywords:** total laparoscopic modified radical hysterectomy, total laparoscopic repair, vaginal cuff dehiscence

## Abstract

Total vaginal cuff dehiscence (VCD) is an important adverse event after hysterectomy. Here, we showed two cases in whom laparoscopic repair of VCD was successful. This procedure is effective, safe, and thus minimally invasive for patients after hysterectomy.

## 1 INTRODUCTION

Vaginal cuff dehiscence is a serious complication following laparoscopic hysterectomy. Here, we present two cases of this complication and the surgical course of each patient. We utilized the total laparoscopic approach, which proved to be effective.

Vaginal cuff dehiscence (VCD) occurs in around 1% of women after laparoscopic hysterectomy and is a rare and serious surgical complication.^1^, ^2^, ^3^ Among reported cases, cases of complete VCD with bowel evisceration are rare. Although there are few reports of total laparoscopic repair for VCD,^4^, ^5^ we performed this procedure in two cases. We reported two cases of complete VCD complicated by bowel evisceration after total laparoscopic modified radical hysterectomy (TLmRH), which was triggered by the first sexual intercourse after primary surgery.

## 2 CASE

### 2.1 Case 1

**Figure 1:**
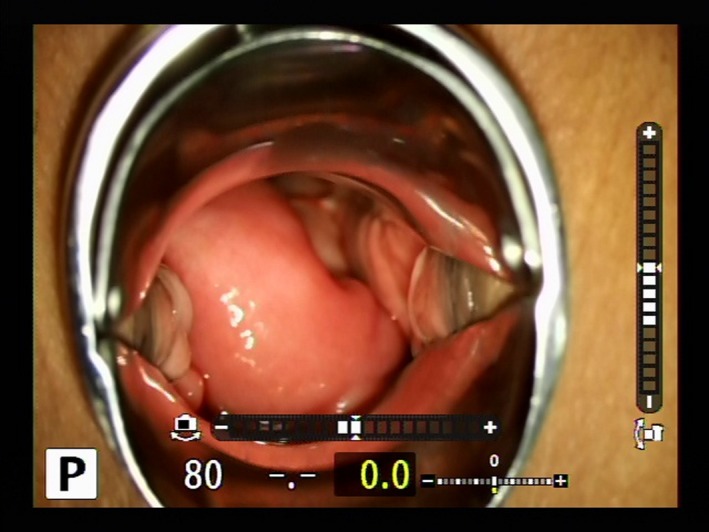
Bowel prolapse through the vagina at emergent visit in the first case

A 47‐year‐old woman, gravida 2, para 2, non‐obese, and with no chronic diseases underwent TLmRH as curative treatment for clinical stage IA endometrial cancer. She had sexual intercourse 6 months after surgery. She noticed organ prolapse during defecation the next day, recognizing something dropped in her vagina (Figure 1).

### 2.2 Case 2

A 33‐year‐old woman, gravida 3, para 3, non‐obese and with no chronic diseases, underwent TLmRH as curative treatment for clinical stage IA1 cervical cancer. Two months later, she presented to our department with abdominal pain and genital bleeding after her first sexual intercourse after surgery from the previous day.

We sutured the vaginal cuff with absorbable sutures during initial surgery. We performed colpotomy with ultrasonic device and monopolar device in both cases.

Both patients were immediately diagnosed with VCD (Figure 1). The prolapsed organ was found to be the intestine and it remained within the vagina without evisceration out of the vagina. The color of the intestine was normal, indicating that there was no ischemia present. After washing of the prolapsed intestine, we pushed back the prolapsed intestine, with sterilized gauze to prevent herniation outside of the vagina until operation. Vaginal approach repair (repair from the vaginal cavity), open approach repair, or laparoscopic approach repair were treatment choices. Suturing from the vagina could shorten vaginal length. To prevent recurrence, we thought it would be better to suture the peritoneum. We already resected the vagina about 2 cm in the initial surgery in both cases; thus, we would like to avoid further shortening. We thought that the open approach should be avoided considering its invasiveness if we could safely avoid this complication laparoscopically. Thus, we initially employed total laparoscopic repair.

**Figure 2:**
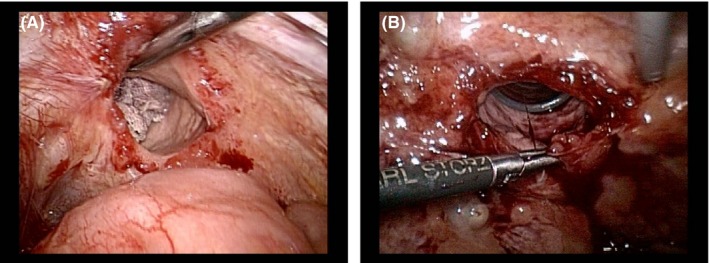
A, The status of vaginal cuff dehiscence observed through laparoscopy in the first case. B, The status of vaginal cuff dehiscence observed through laparoscopy in the second case. The anatomical position between the vaginal cuff and the urinary tract could not be confirmed to be safe for suturing in both cases

**Figure 3:**
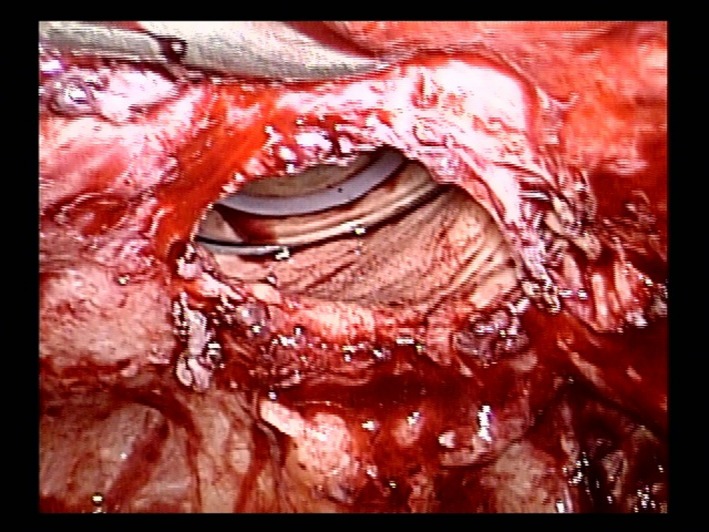
The vaginal tract was revealed carefully at about 1 cm and then re‐sutured to close (in the first case)

**Figure 4:**
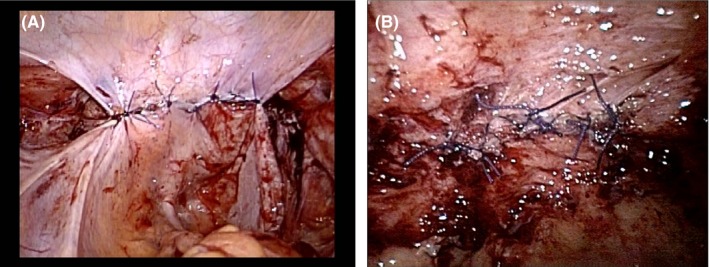
A, The status of the first case after repairing by laparoscopic approach. B, The status of the second case after repairing by laparoscopic approach

The ureter was separated from the paravaginal tissue during initial cancer surgery, losing its normal anatomical position. This may cause ureteral damage during repair (Figure 2). Thus, in order to avoid ureteral injury and to create a tight suture, the vaginal wall was separated by 1.0 cm, to the extent that concrete vaginal cuff suture could be made (Figure 3), and suturing was complete (Figure 4). The peritoneum was sutured to prevent recurrence, hematoma, and infection. The postoperative course was good. The cuff remained intact at 1‐, 2‐, 3‐ and 6‐month examination. We suggested that sexual intercourse is safe 6 months after surgery.

Interviews conducted on these cases after the 6‐month examination revealed that normal sexual intercourse was performed without troubles in both cases.

## 3 DISCUSSION

Although VCD occurs in around 1% of women after laparoscopic hysterectomy,^1^, ^2^, ^3^ it sometimes spontaneously resolves in case of small partial dehiscence without existence of organ evisceration. However, in some VCD cases, organ evisceration, such as small intestine, can occur. In the case of total laparoscopic hysterectomy, the procedure increases the risk for VCD 4.9 times greater than in cases of laparoscopically assisted vaginal hysterectomy, and 9.1 times greater than in cases of the abdominal hysterectomy.^6^ Our two cases had no risk factors regarding aberrant wound healing such as postoperative hematoma, infection, chronic disease, corticosteroid use, and adjuvant chemotherapy/radiotherapy.

The approach to VCD depends on the situation, such as the degree of evisceration, the eviscerated organ, and time lag between the evisceration and hospital visit. In some patients, the presently reported total laparoscopic repair may be useful from the viewpoint of prevention of recurrence and sexual quality because of the depth of vagina. We cannot suture the cuff edge with enough margin, therefore the depth can decrease if we repair through the vaginal approach. The employment of this procedure depends on the situation, especially on the surgeons’ skill. However, we believe that a laparoscopic surgeon with experience in performing extra‐fascial hysterectomy, such as modified radical hysterectomy (Type II),^7^ or radical hysterectomy (Type III),^7^ can perform repairs relatively easily. We recommend that the procedure be selected depending on the situation of the institute.

This laparoscopic procedure is superior to abdominal approach because it is a minimally invasive surgery and is also superior to the vaginal approach in terms of vaginal length, which can influence the quality of sexual intercourse. Moreover, in the laparoscopic approach, surgeons can suture the peritoneum to prevent recurrence and to reduce the risk of hematoma and infection.^3^, ^8^

There are few reports on VCD repair by the total laparoscopic approach^4^, ^5^ describing its safety and efficacy. This is the first and second case in which total laparoscopic repair for VCD after TLmRH was performed. The present report is valuable in indicating a safe technique for VCD repair with laparoscopic approach and improving the sexual quality of life even after extended resection of vaginal wall in primary surgery.

The patients provided informed consent, and the study design was approved by the appropriate ethics review board.

## CONFLICT OF INTEREST

None declared.

## AUTHOR CONTRIBUTION

YS, YI and NR: performed the surgical intervention of the two cases. YI and NR: were in charge of the patients. YS: contributed to write the manuscript. YS, YI, NR and EM: have read and approved the manuscript.
